# NF-κB Inhibition Suppresses Experimental Melanoma Lung Metastasis

**DOI:** 10.26502/jcsct.5079070

**Published:** 2020-08-14

**Authors:** Tomoko Stansel, Samuel A. Wickline, Hua Pan

**Affiliations:** The USF Health Heart Institute, Morsani College of Medicine, University of South Florida, Tampa, FL, USA

**Keywords:** Melanoma, Lung metastasis, NF-κB, siRNA, p5RHH

## Abstract

**Background:**

Although novel therapeutic regimens for melanoma continue to emerge, the best current clinical response rate is still less than 60%. Moreover, antimelanoma treatments contribute to toxicities in other vital organs. In this study, we elucidate the therapeutic advantages of siRNA targeting melanoma NF-κB canonical signaling pathway with a peptide-based gene delivery nanoplex system.

**Methods and Results:**

*In vitro* treatment of melanoma B16-F10 cells was used to demonstrate delivery and efficacy of anti-NF-kB siRNA to cell cytoplasm with a 55 mn peptide-based gene delivery system. NF-κB (p65) knockdown was validated both at mRNA and protein levels by using RT2-PCR, western blot, and immunofluorescence cellular staining. Canonical p65 mRNA was reduced by 82% and p65 protein was reduced by 48%, which differed significantly from levels in control groups. *In vivo* treatment of a melanoma lung metastasis mouse model with 3-serial i.v. injections of p5RHH-p65 siRNA nanoparticles retarded growth of lung metastasis within one week by 76% (p=0.003) as compared to saline control treatments.

**Conclusion:**

Inhibition of melanoma NF-κB (p65) with systemically-delivered siRNA effectively impedes the growth and progression of experimental melanoma lung metastasis.

## 1. Introduction

Malignant melanoma, still a top five common cancer in the United States, exhibits only a 10-20% response rate to conventional chemotherapy [1,2]. Adjuvant immunotherapy with interferon-α and/or interleukin-2 modestly increases the response rate, but excessive toxicity from immunotherapy can outweigh the benefit [2–4]. The FDA-approved agent vemurafenib (PLX4032) used in tumors that harbor the V600E BRAF mutation improves response rates to 48% with extended survival [5]. More recently, immune check point inhibitors combining ipilimumab (anti-CTLA-4) and nivolumab (anti-PD-1) achieve a 58% response rate [6]. However, approximately 90% of patients receiving anti-CTLA-4 [7] and 70% of patients receiving anti-PD-1 [8] or anti-PD-Ll [9] monotherapy experience immune related adverse events. Based on adverse cardiovascular outcomes data from 2018 [10], ~ 34% of the patients developed myocarditis after receiving combination immune checkpoint inhibitors. In view of the modest benefits conferred by currently available therapies in concert with off-target toxicities in vital organs, we propose that a more localized approach to modulating dysregulated driver signaling pathways in the tumor microenvironment may represent a preferred alternative.

The constitutively activated NF-κB signaling pathway is a convergence point for dysregulated cellular signaling pathways in melanoma [11], and plays an important role in melanoma initiation [12], progression [13], invasion [14], metastasis [15], and resistance to chemo-and immunotherapy [16]. Under physiological conditions, NF-κB is sequestered in the cytoplasm as an inactive complex with the inhibitory protein IκB. Upon physiological and/or pathological stimulations, IκB is phosphorylated by the activated IKK complex, resulting in ubiquitination and degradation of IκB. Consequently, NF-κB becomes free to translocate to the nucleus where it initiates the expression of NF-κB dependent genes. Because NF-κB also is critical for proper immune cell function, its systemic inhibition could result in depressed surveillance against cancers and infections [17], indicating that local control at the site of tumor growth would be necessary for optimal clinical benefit and safety.

To that end, we have designed a flexible siRNA nanoplex delivery system comprising an amphipathic peptide (“p5RHH”) capable of condensing siRNA into a stable 55 nm nanoparticle and preclinical validation studies have demonstrated that this peptide-based delivery platform is efficacious in atherosclerosis [18], necrotizing enterocolitis [19], pancreatic cancer [20], cancer angiogenesis [21], ovarian and uterine cancer [22], osteoarthritis [23, 24], and rheumatoid arthritis [25]. Moreover, NF-κB suppression in inflamed targeted tissues remains localized and does not disrupt important signaling actions of NF-κB in off-target tissues/organs [25]. Here we sought to elucidate the benefit of systemically administered p5RHH-p65 siRNA nanoparticles in a mouse model of melanoma lung metastasis and demonstrate that this approach markedly retards the development of melanoma lung metastasis.

## 2. Materials and Methods

### 2.1 Cell culture

Cells were maintained in a humidified atmosphere of 95% air and 5% CO_2_. The B16-F10 cells (CRL-6475, ATCC, Manassas, VA) and B16-F10-eGFP cells were maintained in DMEM (39-2002, ATCC, Manassas. VA) with 10% (v/v) heat-inactivated FBS (10082, life technologies, Thermo Fisher Scientific, Waltham, MA).

### 2.2 p5RHH-p65 siRNA nanoparticle preparation

Preparation of peptide structures for condensation of siRNA into 55 nin particles has been described in prior publications [26–28]. p5RHH peptide was synthesized by GenScript and prepared at 20 mM in molecular biology-grade water (46-000-CI; Corning, New York, NY, USA). siRNAs with or without Cy3 labeling were purchased from Sigma-Aldrich (St Louis, MO, USA) and were prepared at 100 μM in siRNA buffer diluted from 5x siRNA buffer (B-002000-UB-100; Thermo Fisher Scientific, Waltham, MA). For the formulation of nanoparticles, the peptide:siRNA mole ratio was 100:1. For *in vitro* experiments, p5RHH-siRNA nanoparticles were formulated by mixing p5RHH and siRNA in Hanks’ Balanced Salt solution (HBSS) (14025-092; Thermo Fisher Scientific, Waltham, MA) and incubated at 37°C for 40 minutes, followed by albumin stabilization according to prior description [27]. For *in vivo* applications, peptide and siRNA mixtures in HBSS were incubated on ice for 10 minutes prior to intravenous injection at a dose of 0.5 mg siRNA/kg. Prior physical characterization of albumin-coated p5RHH-siRNA nanoparticles revealed a particle size of ~55 nm with polydispersity of 0.282 and ζ-potential of −33.24 mV.

### 2.3 *In vitro* delivery of p5RHH-eGFP siRNA nanoparticles to B16-F10-eGFP cells

B16-F10 eGFP expressing cells were seeded in a Delta TPG Dish (12-071-33, Fisher Scientific, Waltham, MA) at 100,000 cells/ml. Twenty-four hours after seeding, p5RHH-eGFP siRNA nanoparticles with siRNA labeled with Cy3 were incubated with cells at an siRNA concentration of 100 μM. Twenty-four hours later, cells were washed 5 times with PBS with Ca^2+^ and Mg^2+^ (14-040-117, Fisher Scientific, Waltham, MA), before fixation in 4% PFA (50-259-99, Fisher Scientific, Waltham, MA) for 5 minutes at 37°C followed by 5 times wash in PBS with Ca^2+^ and Mg^2+^(14-040-117, Fisher Scientific, Waltham, MA) for confocal microscopic imaging with a Meta 510 (Carl Zeiss, Oberkochen, Germany).

### 2.4 *In vitro* knockdown of NF-κB (p65) in B16-F10 cells

B16-F10 cells were seeded in 6-well plate (TP92006, MidSci, Valley Park, MO) at 100,000 cells/ml. Twenty-four hours after seeding, p5RHH-p65 siRNA nanoparticles were incubated with cells at siRNA concentration of 100 μM for either 24 or 48 hours. After the incubation, cells were washed 5 times with ice cold PBS with Ca^2+^ and Mg^2+^ (14-040-117, Fisher Scientific, Waltham, MA) before mRNA or protein extraction.

### 2.5 RT^2^-PCR

Total RNA from B16-F10 cells was isolated using an RNeasy minikit (74104; Qiagen, Hilden, Germany). By reverse transcription with an RT^2^ first-strand kit (330401; Qiagen, Hilden, Germany), RNA (1 μg) was used to synthesize cDNA. Real-time PCR analysis was performed on an ABI 7300 system (Thermo Fisher Scientific, Waltham, MA) with RT^2^ first SYBR green/ROX PCR master mix (330530; Qiagen, Hilden, Germany). Specific primers for each gene were purchased from Qiagen. Genes of interest were normalized to mouse β-actin.

### 2.6 Western blot

Radioimmunoprecipitation assay (RIPA) buffer (R0278-500ML; Sigma-Aldrich. St. Louis, MO) with 1 tablet protease inhibitors (4906837001; Sigma-Aldrich. St. Louis, MO) per 10 mL RIPA buffer and phenylmethylsulfonyl fluoride (8553; Cell Signaling Technology, Danvers, MA) at a final concentration of 1 mM was used to extract proteins from B16-F10 cells. Briefly, cells were disrupted in lysis buffer and protein lysates were obtained by centrifugation for 10 minutes at 12,000 × *g* at 4°C. Protein concentration was quantified with BCA protein assay (23225; Thermo Fisher Scientific, Waltham, MA). Under reducing conditions, equivalent amounts of total protein were fractionated using sodium dodecyl sulfate polyacrylamide-gel electrophoresis. Membranes were probed with NF-κB p65 (D14E12) XP^®^ Rabbit mAb (1:1,000 dilution, 8242S; Cell Signaling Technology, Danvers, MA) and anti-beta actin antibody (1:1,000 dilution, ab8227; Abeam, Cambridge, MA). Membranes were washed and incubated with secondary antibody anti-rabbit HRP (1:10,000 dilution, sc-2313; Santa Cruz Biotechnology). Bands were visualized with Pierce ECL Western blotting substrate (32106; Thermo Fisher Scientific, Waltham, MA) with a ChemiDoc MP (Bio-Rad Laboratories, Hercules, CA, USA). Knockdown of proteins was quantified with ImageJ (National Institutes of Health, Bethesda, MD, USA).

### 2.7 Immunofluorescence cellular staining

B16-F10 cells were seeded in 6-well plate (TP92006, MidSci, Valley Park, MO) at 100,000 cells/ml on circular cover slips (12CIR-1, Thermo Fisher Scientific, Waltham, MA). Twenty-four hours after seeding, p5RHH-p65 siRNA nanoparticles were incubated with cells at an siRNA concentration of 100 μM. Twenty-four hours later, cells were washed 5 times with PBS with Ca^2+^ and Mg^2+^ (14-040-117, Fisher Scientific, Waltham, MA), before fixation in 4% PFA (50-259-99, Fisher Scientific, Waltham, MA) for 5 minutes at 37°C followed by 5 times wash with PBS with Ca^2+^ and Mg^2+^ (14-040-117, Fisher Scientific, Waltham, MA), and incubation with rabbit anti-NFκB p65 (D14E12) XP (1:200 dilution, 8242S; Cell Signaling Technology, Danvers, MA) overnight at 4°C and then incubation in goat anti-rabbit IgG H&L (Alexa Fluor^®^ 594) (1:500 dilution, abl50080; Abcam, Cambridge, MA) for 30 minutes at room temperature. Images were acquired with an Olympus microscope all at the same exposure times and settings.

### 2.8 Lung metastasis induction, treatment, and quantification

Male C57BL/C mice were injected with half million B16-F10 cells (Day 0) in PBS. p5RHH-p65 siRNA nanoparticles at 0.5 mg/kg or saline (control) was injected on days 4, 5, and 6. Twenty-four hours after the last treatment, mice were euthanized and lungs collected for tumor metastasis response. To quantity metastases, the lungs were blotted dry and imaged at 10X with a Leica dissecting microscope. The digitized images were processed by ImageJ for lung metastasis quantification. Briefly, the images were converted to 8-bit and brightness/contrast adjusted, before threshold for lung metastasis quantification.

### 2.9 Animal study approval

Animal experiments were completed in compliance with US federal laws and in accordance with Washington University Division of Comparative Medicine guidelines. The animal protocol is reviewed annually and approved by the Washington University Animal Studies Committee.

### 2.10 Statistics

Results are expressed as mean ± standard error of mean (SEM). Two-sided t-testing and One-Way ANOVA with Scheffe test were used. Statistical significance of differences was attributed at p < 0.05.

## 3. Results

### 3.1 *In vitro* delivery of siRNA to the melanoma cells

**Figure 1:**
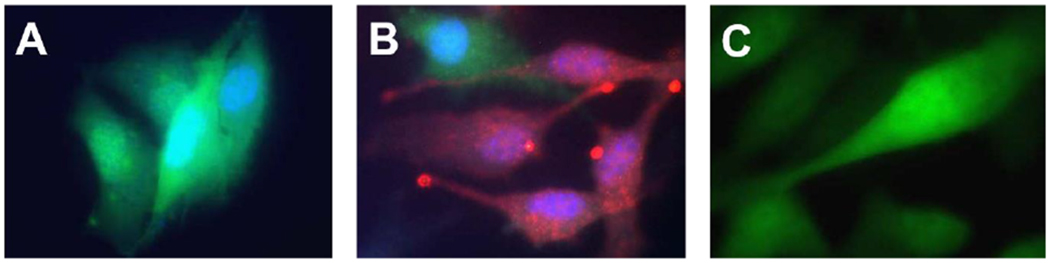
Delivery of siRNA to melanoma cells using p5RHH peptide nanoparticles. Representative confocal images of: A) B16-F10 eGFP cells, (B) B16-F10 eGFP cells treated with p5RHH-Cy3-labeled anti-eGFP siRNA nanoparticles, and (C) B16-F10 eGFP cells treated with free Cy3-labeled eGFP siRNAs. Green: eGFP expression signal; Red: Cy3 labeled peptide nanoparticles; Blue: nucleus.

To confirm the capability of peptide-based nanostructures for delivering siRNA to melanoma (B16-F10) cells, B16-F10 cells with stable eGFP expression were used for visualization of Cy3-labeled eGFP siRNA delivery by p5RHH (p5RHH-Cy3-labeled Egfp siRNA nanoparticles). Groups were: 1) B16-F10 eGFP cells untreated, 2) B16-F10 eGFP cells treated with p5RHH-Cy3-labeled eGFP siRNA nanoparticles, or 3) B16-F10 eGFP cells treated with free Cy3-labeled eGFP siRNA for 24 hours before imaging. Under confocal fluorescence imaging, B16-F10 eGFP cells exhibited green florescence (Figure 1A). After p5RHH-Cy3-labeled eGFP siRNA nanoparticle treatment, cells with eGFP siRNAs delivered by the nanoparticles turned red (Figure 1B), which demonstrated 1) Cy3-labeled eGFP siRNAs (red) were delivered to the cytoplasm of B16-F10 eGFP cells, and 2) eGFP expression had been knocked down (lack of green signal) by the siRNAs delivered by the nanoparticles. However, incubation of the cells with free Cy3-labeled eGFP siRNAs remained green (Figure 1C) suggesting that free siRNAs were not capable of entering cells as expected.

### 3.2 *In vitro* NF-κB (p65) knockdown by p5RHH-p65 siRNA nanoparticles

**Figure 2:**
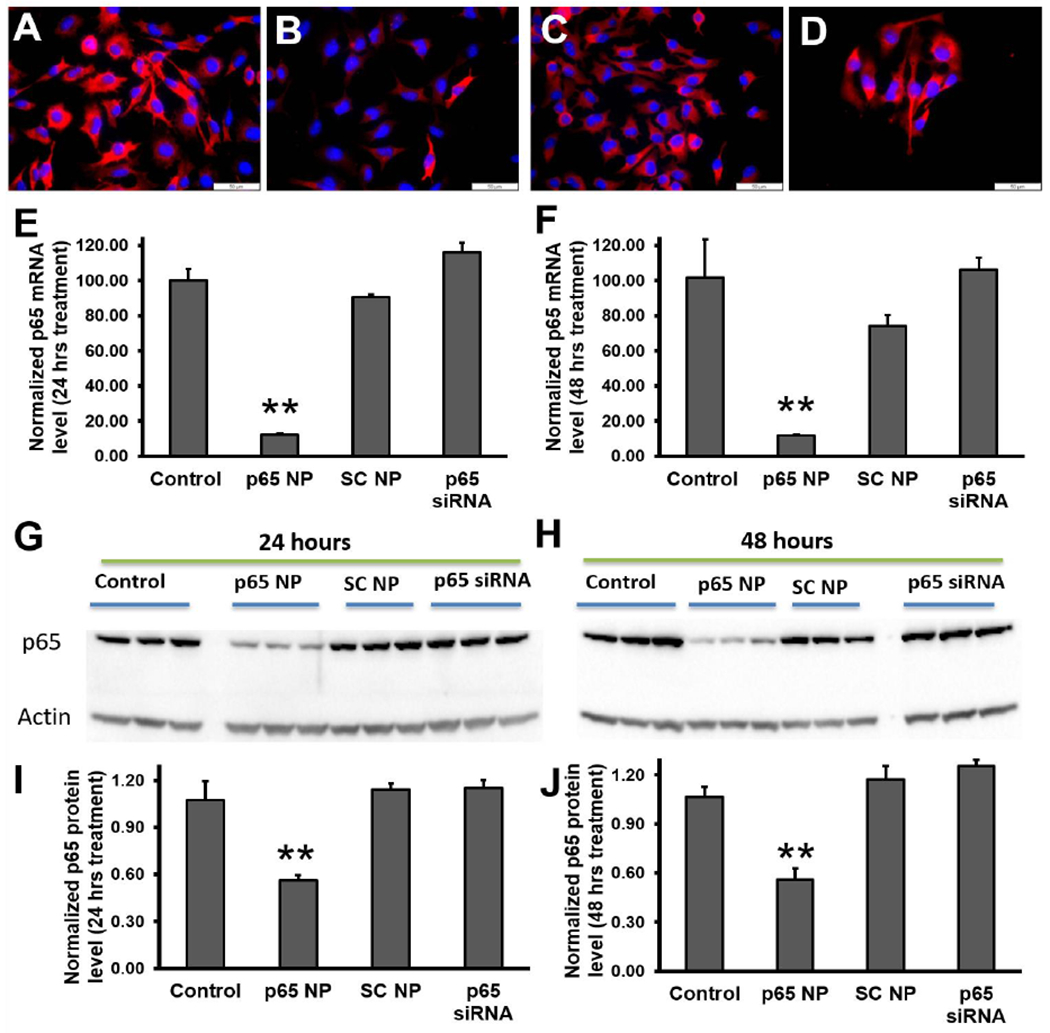
NF-κB (p65) knockdown in melanoma cells by using p5RHH-p65 siRNA nanoparticles. Reprehensive fluorescence images of p65 staining on B16-F10 cells (A), treated with p5RHH-p65 siRNA nanoparticles (B), treated with p5RHH-scrambled siRNA nanoparticles (C), or treated with free p65 siRNAs (D). RT2-PCR of p65 mRNA level knockdown at 24 hours (E) and 48 hours (F) post treatment, respectively. Western blot (G-H) and the quantifications (I and J) showing significant knockdown only when p65 siRNAs were delivered by p5RHH. **: p<0.01, n=3.

To evaluate the knockdown of p65 in melanoma cells (B16-F10) with p5RHH-p65 siRNA nanoparticles, the following four experimental groups were conducted: 1) control (no treatment), 2) p5RHH-p65 siRNA nanoparticles (p65 NP), 3) p5RHH-scrambled siRNA nanoparticles (SC NP), and 4) free p65 siRNA (p65 siRNA). The knockdown was evaluated for both mRNA and protein suppression. The immunofluorescence staining results (Figure 2A–D) suggested that only p5RHH-p65 siRNA nanoparticles treatment reduced p65 protein level in the melanoma cells. For mRNA analysis, by 24 hours after p5RHH-p65 siRNA nanoparticle treatment p65 mRNA was reduced by 82% and was significantly different than that of control cells with no treatment (p=6.38e^−8^), cells treated with p5RHH-scrambled siRNA nanoparticles (p=1.60e^−7^), and cells treated with free p65 siRNA (p=1.72e^−8^) (Figure 2E). 48 hours after p5RHH-p65 siRNA nanoparticle treatment, p65 mRNA maintained reduction at 12% that of control cells and was significantly different than control cells with no treatment (p=1.16e^−4^), cells treated with p5RHH-scrambled siRNA nanoparticles (p=0.001), and cells treated with free p65 siRNA (p=7.95e^−5^) (Figure 2F). As for p65 protein level, 24 hours after p5RHH-p65 siRNA nanoparticle treatment, p65 protein was reduced by 48% and was significantly different than that of control cells with no treatment (p=0.006), cells treated with p5RHH-scrambled siRNA nanoparticles (p=0.003), and cells treated with free p65 siRNA (p=0.002) (Figure 2G and I). 48 hours after p5RHH-p65 siRNA nanoparticle treatment, p65 mRNA was still reduced by 48% and was significantly different than that of control cells with no treatment (p=0.004), cells treated with p5RHH-scrambled siRNA nanoparticles (p=0.001), and cells treated with free p65 siRNA (p=4.43 e^−4^) (Figure 2H and J). The results demonstrate that only p5RHH-p65 siRNA nanoparticles treatment significantly reduced p65 in melanoma cells both at mRNA and protein levels.

### 3.3 *In vivo* p5RHH-p65 siRNA nanoparticles inhibit lung metastasis

**Figure 3:**
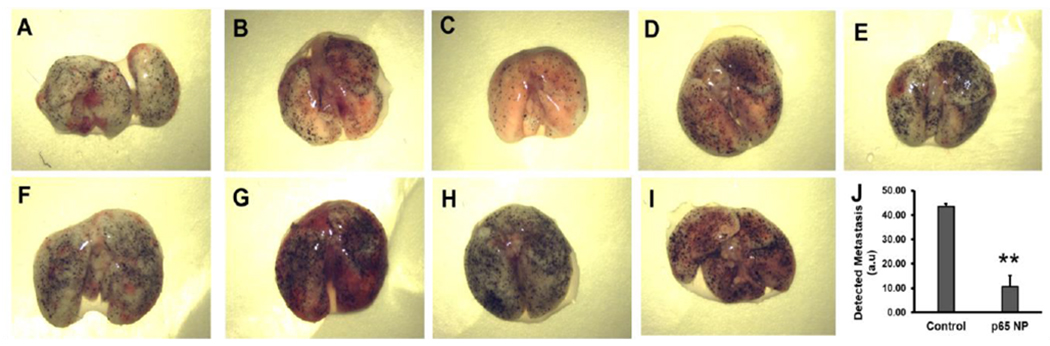
p5RHH-p65 siRNA nanoparticles inhibit the growth of melanoma lung metastases. (A-E) Lungs from the mice with p5RHH-p65 siRNA nanoparticles treatment (n=5) and (F-I) lungs from the mice with saline treatment (n=4). J. quantification of lung metastasis indicate lung metastasis progress was inhibited by the nanoparticle treatment (p=0.003). Results were presented as Mean±SEM.

To assess the effects of p5RHH-p65 siRNA nanoparticles treatment on lung metastasis, we employed a standard melanoma lung metastasis mouse model, which develops significant lung metastasis within 7 days after i.v. injection of half million B16-F10 cells. Given the fact that this is a very aggressive lung metastasis model, at day 0, mice received i.v. injection of B16-F10 cells and the treatment was introduced on day 4 for three consecutive days and the mice were sacrificed on day 7. The lungs from the p5RHH-p65 siRNA nanoparticles treated mice (Figure 3A–E) exhibited a 76% reduction in lung metastasis (10.54 ± 4.73) as compared to those in the saline treated mice (Figure 3F–I) (43.57 ± 1.09) (p=0.003).

## 4. Discussion

Following the initial description of endogenous RNA silencing machinery, the possibility that exogenously synthesized siRNA might serve the same purpose to inhibit RNA translation by engaging the RISC complex was demonstrated [29, 30]. Much has been discovered about the nature of the RNA molecule itself regarding modifications that stabilize and improve efficiency [31–33], but the search for a broadly applicable nonviral delivery platform remains a key to clinical adoption and utility. Traditional transfection agents including cationic lipids and polymers manifest high efficiency but can elicit cytotoxicity and are routinely sequestered in the liver. The current p5RHH peptide-nucleotide nanoplex delivery approach is agnostic to the nucleotide cargo selected and has been adopted in many labs working in collaboration on various disease substrates beyond the liver such as cancer [20–22], atherosclerosis [18, 34, 35], necrotizing enterocolitis [19], and arthritis [23–25]. The p5RHH protects the siRNA in circulation, while promoting a controlled sequence of cell entry, endosomal escape, and cytoplasmic siRNA release [26–28]. The interaction between nucleotide and peptide is initially electrostatic, but importantly an exothermic process of strong hydrogen bonding takes place between the histidines and nucleic acids to markedly stabilize the polyplex, as shown elegantly by Chou et al. [36]. Particle disassembly mandates an absolute requirement for an acidic pH such that particles are highly stable in circulation at neutral pH [26, 27]. Endosomal acidification at pH<5 results in complete protonation of the imidazole groups (pKa ~ 6.2) on histidine residues that disrupts strong hydrogen bonding, causing nanoplex disassembly, release of the nucleotides, and freeing of the p5RHH moiety. The free p5RHH peptide moiety is released locally at sufficient concentrations to disrupt the endosomal membrane and release the siRNA, whereas in circulation at physiological pH it cannot disassemble and disrupt other cell membranes [37]. Upon release from the endosome, the peptide is rapidly diluted in cytoplasm and exerts no untoward cell lytic action.

The present data demonstrate that inhibition of NF-κB (p65) expression is effective in controlling the growth of experimental melanoma lung metastasis. We have shown previously that NF-κB can be inhibited selectively in inflammatory pathologies such as rheumatoid arthritis without affecting adaptive or innate immune functioning and without inducing an immune response to the agent itself [25]. Moreover, NF-κB is not suppressed in other organs or tissues after i.v. injection because the peptide nanoplexes do not cross vascular territories with normal barrier function a contrasted with permeation of tumor vasculature by the endothelial permeability and retention (EPR) effect [38].

We suggest that suppression of canonical NF-κB signaling might be most useful as an adjunctive therapy in combination with other agents to augment anti-tumor activity or perhaps reduce the need for higher doses of more toxic agents. It also might be possible that inhibiting both canonical and non-canonical NF-κB signaling will provide additional benefit for interdicting melanoma lung metastasis, winch could be tested easily by combining siRNAs in the same nanoplex since the formed particles are agnostic to the actual nucleotide sequence. In any event, these results confirm an important role for NF-κB signaling in the progression of melanoma lung metastasis and illustrate a simple and flexible systemic therapeutic strategy for its local control.

## References

[1] Sondak VK, Ross MI, Schuchter LM. Early stage (I, II, III) melanoma. Current treatment options in oncology 2 (2001): 183–191. 12057118 10.1007/s11864-001-0032-6

[2] Algazi AP, Soon CW, Daud AI. Treatment of cutaneous melanoma (): current approaches and future prospects. Cancer management and research 2 (2010): 197–211. 21188111 10.2147/CMR.S6073 PMC3004577

[3] de Gast GC, Batchelor D, Kersten MJ, Temozolomide followed by combined immunotherapy with GM-CSF, low-dose IL2 and IFN alpha in patients with metastatic melanoma. British journal of cancer. 88 (2003): 175–80. 12610499 10.1038/sj.bjc.6600717 PMC2377058

[4] Meric JB, Rixe O, Kliayat D. Metastatic malignant melanoma. Drags Today (Bare). 39 (2003): 17–38. 14988744

[5] Heakal Y, Kester M, Savage S. Vemurafenib (PLX4032): an orally available inhibitor of mutated BRAF for the treatment of metastatic melanoma. Ann Phannacother 45(2011): 1399–1405. 10.1345/aph.1Q363 22028422

[6] Hastings MPRaKT. Chapter 9 Immune Checkpoint Inhibitors in the Treatment of Melanoma: From Basic Science to Clinical Application Cutaneous Melanoma: Etiology and Therapy (2017). 29461774

[7] Hodi FS, O’Day SJ, McDermott DF, Improved survival with ipilimumab in patients with metastatic melanoma. N Engl J Med 363 (2010): 711–723. 20525992 10.1056/NEJMoa1003466 PMC3549297

[8] Topalian SL, Hodi FS, Brahmer JR, Safety, activity, and immune correlates of anti-PD-1 antibody in cancer. N Engl J Med 366 (2012): 2443–2454. 22658127 10.1056/NEJMoa1200690 PMC3544539

[9] Brahmer JR, Tykodi SS, Chow LQ, Safety and activity of anti-PD-L1 antibody in patients with advanced cancer. N Engl J Med 366 (2012): 2455–2465. 22658128 10.1056/NEJMoa1200694 PMC3563263

[10] Mahmood SS, Fradley MG, Cohen JV, Myocarditis in Patients Treated With Immune Checkpoint Inhibitors. J Am Coll Cardiol 71 (2018): 1755–1764. 29567210 10.1016/j.jacc.2018.02.037 PMC6196725

[11] Amiri KI, Richmond A. Role of nuclear factor-kappa B in melanoma. Cancer metastasis reviews 24 (2005): 301–13. 15986139 10.1007/s10555-005-1579-7 PMC2668255

[12] Wang CY, Mayo MW, Korneluk RG, NF-kappaB antiapoptosis: induction of TRAF1 and TRAF2 and c-IAP1 and c-IAP2 to suppress caspase-8 activation. Science. New York, NY 281 (1998): 1680–1683. 10.1126/science.281.5383.1680 9733516

[13] Yang J, Splittgerber R, Yull FE, Conditional ablation of Ikkb inhibits melanoma tumor development in mice. The Journal of clinical investigation 120 (2010): 2563–2574. 20530876 10.1172/JCI42358 PMC2898608

[14] Beshir AB, Ren G, Magpusao AN, Raf kinase inhibitor protein suppresses nuclear factor-kappaB-dependent cancer cell invasion through negative regulation of matrix metalloproteinase expression. Cancer letters 299 (2010): 137–149. 20855151 10.1016/j.canlet.2010.08.012 PMC2967644

[15] Wu FH, Yuan Y, Li D, Endothelial cell-expressed Tim-3 facilitates metastasis of melanoma cells by activating the NF-kappaB pathway. Oncology reports 24 (2010): 693–699. 20664975

[16] Ueda Y, Richmond A. NF-kappaB activation in melanoma. Pigment cell research / sponsored by the European Society for Pigment Cell Research and the International Pigment Cell Society 19 (2006): 112–124. 10.1111/j.1600-0749.2006.00304.x PMC2668252 16524427

[17] Smale ST. Selective transcription in response to an inflammatory stimulus. Cell 140 (2010): 833–844. 20303874 10.1016/j.cell.2010.01.037 PMC2847629

[18] Pan H, Palekar RU, Hou KK, Anti-JNK2 peptide-siRNA nanostructures improve plaque endothelium and reduce thrombotic risk in atherosclerotic mice. Int J Nanomedicine 13 (2018): 5187–5205. 30233180 10.2147/IJN.S168556 PMC6135209

[19] MohanKumar K, Namachivayam K, Song T, A murine neonatal model of necrotizing enterocolitis caused by anemia and red blood cell transfusions. Nat Commun 10 (2019): 3494. 31375667 10.1038/s41467-019-11199-5 PMC6677753

[20] Strand MS, Krasnick BA, Pan H, Precision delivery of RAS-inhibiting siRNA to KRAS driven cancer via peptide-based nanoparticles. Oncotarget 10 (2019): 4761–4775. 31413817 10.18632/oncotarget.27109 PMC6677667

[21] Kabir AU, Lee TJ, Pan H, Requisite endothelial reactivation and effective siRNA nanoparticle targeting of Etv2/Er71 in tumor angiogenesis. JCI Insight 3 (2018). 10.1172/jci.insight.97349 PMC5931123 29669933

[22] Mills KA, Quinn JM, Roach ST, p5RHH nanoparticle-mediated delivery of AXL siRNA inhibits metastasis of ovarian and uterine cancer cells in mouse xenografts. Sci Rep 9 (2019): 4762. 30886159 10.1038/s41598-019-41122-3 PMC6423014

[23] Yan H, Duan X, Pan H, Suppression of NF-kappaB activity via nanoparticle-based siRNA delivery alters early cartilage responses to injury. Proc Natl Acad Sci U S A 113 (2016): E6199–E6208. 27681622 10.1073/pnas.1608245113 PMC5068304

[24] Yan H, Duan X, Pan H, Development of a peptide-siRNA nanocomplex targeting NF- kappaB for efficient cartilage delivery. Sci Rep 9 (2019): 442. 30679644 10.1038/s41598-018-37018-3 PMC6345850

[25] Zhou HF, Yan H, Pan H, Peptide-siRNA nanocomplexes targeting NF-kappaB subunit p65 suppress nascent experimental arthritis. The Journal of clinical investigation 124 (2014): 4363–4374. 25157820 10.1172/JCI75673 PMC4191028

[26] Hou KK, Pan H, Lanza GM, Melittin derived peptides for nanoparticle based siRNA transfection. Biomaterials 34 (2013): 3110–3119. 23380356 10.1016/j.biomaterials.2013.01.037 PMC3578292

[27] Hou KK, Pan H, Ratner L, Mechanisms of nanoparticle-mediated siRNA transfection by melittin-derived peptides. ACS nano 7 (2013): 8605–8615. 24053333 10.1021/nn403311c PMC4013830

[28] Zhou HF, Yan H, Pan H, Peptide-siRNA nanocomplexes targeting NF-kappaB subunit p65 suppress nascent experimental arthritis. The Journal of clinical investigation 124 (2014): 4363–4374. 25157820 10.1172/JCI75673 PMC4191028

[29] Fire A, Xu S, Montgomery MK, Potent and specific genetic interference by double-stranded RNA in Caenorhabditis elegans. Nature 391 (1998): 806–811. 9486653 10.1038/35888

[30] Elbashir SM, Harborth J, Lendeckel W, Duplexes of 21-nucleotide RNAs mediate RNA interference in cultured mammalian cells. Nature 411 (2001): 494–498. 11373684 10.1038/35078107

[31] Terrazas M, Kool ET. RNA major groove modifications improve siRNA stability and biological activity. Nucleic Acids Res 37 (2009): 346–353. 19042976 10.1093/nar/gkn958 PMC2632910

[32] Kenski DM, Butora G, Willingham AT, siRNA-optimized Modifications for Enhanced *In Vivo* Activity. Mol Ther Nucleic Acids 1 (2012): e5. 23344622 10.1038/mtna.2011.4 PMC3381598

[33] Dar SA, Thakur A, Qureshi A, siRNAmod: A database of experimentally validated chemically modified siRNAs. Sci Rep 6 (2016): 20031. 26818131 10.1038/srep20031 PMC4730238

[34] Vendrov AE, Stevenson MD, Alahari S, Attenuated Superoxide Dismutase 2 Activity Induces Atherosclerotic Plaque Instability During Aging in Hyperlipidemic Mice. J Am Heart Assoc 6 (2017). 10.1161/JAHA.117.006775 PMC5721769 29079564

[35] Lozhkin A, Vendrov AE, Pan H, NADPH oxidase 4 regulates vascular inflammation in aging and atherosclerosis. J Mol Cell Cardiol 102 (2017): 10–21. 27986445 10.1016/j.yjmcc.2016.12.004 PMC5625334

[36] Chou ST, Hom K, Zhang D, Enhanced silencing and stabilization of siRNA polyplexes by histidine-mediated hydrogen bonds. Biomaterials 35 (2014): 846–855. 24161165 10.1016/j.biomaterials.2013.10.019 PMC3920840

[37] Pan H, Myerson JW, Ivashyna O, Lipid membrane editing with peptide cargo linkers in cells and synthetic nanostructures. Faseb J 24 (2010): 2928–2937. 20335225 10.1096/fj.09-153130 PMC2909291

[38] Rosenblum D, Gutkin A, Dammes N, Peer D. Progress and challenges towards CRISPR/Cas clinical translation. Adv Drug Deliv Rev (2020). 10.1016/j.addr.2020.07.004 32659256

